# Functions of RNAi Pathways in Ribosomal RNA Regulation

**DOI:** 10.3390/ncrna10020019

**Published:** 2024-03-29

**Authors:** Aleksei S. Shatskikh, Elena A. Fefelova, Mikhail S. Klenov

**Affiliations:** 1 Koltzov Institute of Developmental Biology, Russian Academy of Sciences, 26 Vavilov Street, 119334 Moscow, Russia; shackih@yandex.ru; 2 Institute of Molecular Genetics, Russian Academy of Sciences, 2 Kurchatov Sq., 123182 Moscow, Russia; 3 RNA Therapeutics Institute, University of Massachusetts Chan Medical School, 368 Plantation Street, Worcester, MA 01605, USA

**Keywords:** rDNA, rRNA, transcription, RNA interference, small RNAs, gene regulation, piRNA, nucleolar dominance

## Abstract

Argonaute proteins, guided by small RNAs, play crucial roles in gene regulation and genome protection through RNA interference (RNAi)-related mechanisms. Ribosomal RNAs (rRNAs), encoded by repeated rDNA units, constitute the core of the ribosome being the most abundant cellular transcripts. rDNA clusters also serve as sources of small RNAs, which are loaded into Argonaute proteins and are able to regulate rDNA itself or affect other gene targets. In this review, we consider the impact of small RNA pathways, specifically siRNAs and piRNAs, on rRNA gene regulation. Data from diverse eukaryotic organisms suggest the potential involvement of small RNAs in various molecular processes related to the rDNA transcription and rRNA fate. Endogenous siRNAs are integral to the chromatin-based silencing of rDNA loci in plants and have been shown to repress rDNA transcription in animals. Small RNAs also play a role in maintaining the integrity of rDNA clusters and may function in the cellular response to rDNA damage. Studies on the impact of RNAi and small RNAs on rRNA provide vast opportunities for future exploration.

## 1. Introduction

### 1.1. Ribosomal DNA Loci and Ribosome Biogenesis

In living cells, proteins are synthesized by ribosomes, the central components of which are ribosomal RNAs (rRNAs). Cells in an active growth phase produce thousands of ribosomes per minute [1]. To enable this productivity, genomes contain hundreds of tandemly repeated rRNA genes organized into rDNA clusters, also referred to as Nucleolar Organizing Regions (NORs) [2]. In eukaryotes, specialized RNA polymerase I (Pol I) transcribes rRNA genes, generating polycistronic precursor pre-rRNA transcripts (47S pre-rRNA in mammals). These transcripts include 18S, 5.8S, and 28S rRNA sequences, as well as external and internal transcribed spacers (ETS, ITS), which are eliminated during processing. Individual rRNA genes are separated by intergenic spacer regions (IGS), containing various regulatory elements. Another ribosome component, 5S rRNA, is synthesized by RNA polymerase III (Pol III) and, in most eukaryotes, is encoded by distinct, separately located clusters [3].

Most stages of eukaryotic ribosome biogenesis occur within the nucleolus, encompassing processes such as rDNA transcription, rRNA processing, modification, folding, maturation, and the assembly of pre-ribosomal particles with ribosomal proteins. These intricate steps are meticulously coordinated by specific enzymes and small nucleolar RNAs (snoRNAs), which guide rRNA modifications. Following these events, pre-ribosomal subunits are transported to the cytoplasm, where additional maturation takes place. Several nucleolar and cytoplasmic surveillance machinery control the quality of rRNA and the assembly of pre-ribosome intermediates (reviewed by [4]).

The level of rRNA synthesis is extremely flexible and depends on environmental conditions, cell type, and the stage of the cell cycle. This regulation is achieved by various metabolic and signaling pathways, including mTOR and MYC (reviewed by [5,6]) as well as by long non-coding RNAs (reviewed by [7,8]). In turn, the level of rRNA and ribosome production defines the global level of protein synthesis and influences cell growth, development, and differentiation. For instance, inhibition of rDNA transcription can induce cell differentiation in mice and human cells, while rDNA activation maintains stemness [9,10,11,12]. Abnormalities in rRNA production are linked to various human diseases (reviewed by [13,14,15]), including Treacher Collins syndrome, Börjeson–Forssman–Lehmann syndrome, characterized by developmental defects and mental retardation, and some neurodegenerative diseases. An increased rRNA production is often associated with cancer progression.

In addition to specific regulatory mechanisms, the rDNA locus undergoes epigenetic regulation following principles common to other genes in eukaryotes. In mammalian cells, silent rRNA genes often, though not universally, are characterized by methylated DNA, along with repressive histone marks such as H3K9me3, H4K20me3, and H3K27me3, coupled with deacetylated histones (for a review, see [16,17]), whereas the bodies of actively transcribed rRNA genes are thought to be free of nucleosomes [18,19]. Nucleosome remodeling machinery also plays a pivotal role in controlling rDNA transcription initiation by shifting the promoter-associated nucleosome between active and repressive positions [20].

Due to their repetitive structure, rDNA loci are prone to intrachromosomal recombination, leading to the loss of some rDNA copies. Accordingly, there are cellular mechanisms aimed at restoring the number of rDNA copies through homology-dependent repair (reviewed in [21,22]). These dynamic changes cause the number of rDNA units to vary greatly between individuals. For instance, fruit fly *Drosophila melanogaster* is reported to have an rDNA copy number ranging from 80 to 600 [23], the range of copies for nematode *Caenorhabditis elegans* is approximately 30 to 250 [24], and in humans, the range extends from 14 to 410 [25] or from 61 to 1590 [26]. Moreover, rDNA copy number varies even within cells of the same organism and depends on age [27,28,29].

Remarkably, only a fraction of rRNA genes in eukaryotic genomes are actively transcribed, while others are typically reversibly inactivated [30,31,32]. Some individual NORs can be completely repressed [33], while within active NORs, some rDNA units are silent and bear repressive epigenetic marks as has been shown in human and *Drosophila* cells [34,35,36,37]. This regulation appears to ensure the necessary level of rRNA synthesis despite variations in rDNA copy number and also may help to prevent recombination between rDNA repeats, repress abnormal rDNA units, or select specific rDNA variants for transcription. Interestingly, variant rRNA alleles containing single-nucleotide polymorphisms exhibit tissue-specific expression in mice [26].

### 1.2. Small RNA Pathways

Small RNAs associated with Argonaute (AGO) proteins serve as sequence-specific guides in nucleic acid-based processes collectively referred to as RNA interference (RNAi). Small RNAs direct the AGO complex toward complementary nucleic acids via Watson–Crick base-pairing, resulting in gene repression through various mechanisms, including RNA cleavage, translation inhibition, or repression of target transcription in the nucleus (reviewed in [38,39]).

Historically, three major classes of small RNAs have been discerned: small interfering RNA (siRNA), microRNA (miRNA), and Piwi-interacting RNA (piRNA). These classes are distinguished by their biogenesis, origins, and functionalities [40]. siRNAs may stem from either exogenous double-stranded RNA (dsRNA) or endogenous hairpin RNA precursors, undergoing processing through the RNase Dicer with accessory proteins [41]. Notably, in some organisms, the biogenesis of certain siRNA subtypes requires RNA-dependent RNA polymerases (RdRp), which can synthesize dsRNA on a single-stranded RNA template.

miRNAs emerge from endogenous transcripts featuring specific hairpin loop structures, determining subsequent processing and defining the termini of the mature miRNA. Typically, miRNAs regulate gene expression at the post-transcriptional level by binding to target mRNAs, leading to translational repression or mRNA degradation (reviewed in [42,43]).

The third class, piRNAs, unlike other small RNAs, are produced in a Dicer-independent manner from single-stranded transcripts loaded into Piwi subfamily Argonaute proteins (reviewed in [44]). In most animals, including *Drosophila* and mammals, piRNA biogenesis relies on two conserved piRNA-specific mechanisms: phased cleavage by the Zucchini endonuclease (phasing) and ping-pong amplification. *C. elegans* stands out as an exception, where individual piRNAs (known as 21U RNAs) are transcribed from mini-genes. The Piwi-piRNA pathway plays a pivotal role in transposon silencing, safeguarding the germline genome, and also serves other biologically important functions in animals.

Subsequently, additional categories of small RNAs have been characterized in eukaryotes, including those that originated from highly abundant cellular transcripts such as tRNA and rRNA. Among them, tRNA-derived fragments (tRFs), produced from pre-tRNAs or mature tRNAs, have demonstrated clear biological functions in cancers and stress response, solidifying their status as important regulatory non-coding RNAs (reviewed in [45]). rDNA-derived small RNAs have been described in various organisms, including plants [46,47,48,49], fungi [50,51,52,53,54], fruit flies [55], *C. elegans* [56,57], mice, and humans [58,59,60]. In addition to small RNAs originating from mature rRNAs, commonly denoted as rRFs (rRNA-derived fragments), small RNAs can emerge from pre-rRNAs, intergenic spacer regions (IGS), and antisense rDNA transcripts. These RNAs form complexes with various AGOs and, depending on their biogenesis and interacting proteins, may belong to siRNAs [56], miRNAs [55,61], piRNAs [62], and other types like phased small interfering RNAs (phasiRNAs) originating from 28S and 5.8S rRNAs in plants [63]. An expanding body of evidence suggests that rRFs might fulfill biologically relevant functions, including gene regulation unrelated to rRNA and ribosome biogenesis (reviewed in [45]). However, in this review, we aim to consolidate existing data concerning the potential roles of small RNAs and RNAi mechanisms in the regulation of rRNA itself.

## 2. The Role of siRNAs in the Nucleolar Dominance

Nucleolar dominance is an epigenetic phenomenon in which rRNA genes derived from one parent are actively expressed, while the genes inherited from the other parent undergo transcriptional silencing independent of maternal or paternal effects [64]. This phenomenon occurs at the level of entire NORs and is manifested in the fact that some rDNA loci are completely silent [65,66]. Nucleolar dominance is observed in animal and plant interspecific hybrids, for example, in *Arabidopsis suecica*, a hybrid of *A. thaliana* and *A. arenosa* species [67]. A NOR comprising hundreds of rRNA genes from *A. thaliana* is repressed, while rRNA genes from the *A. arenosa* NOR are transcribed [65,68]. There is reason to believe that nucleolar dominance reflects a general mechanism of rRNA expression control, also observed in non-hybrid organisms, providing rRNA level adjustment depending on the need for ribosomes and protein synthesis. The transcriptional status of rRNA genes subject to nucleolar dominance is tissue-specific [69] and can change during development [70].

The underlying nucleolar dominance transcriptional silencing of rDNA clusters in plants is achieved by an increased level of DNA methylation and changes in histone marks, particularly H3K9 methylation and histone deacetylation caused by the histone deacetylase HDA6 [71,72,73]. This silencing also requires the methylcytosine-binding proteins MBD6 and MBD10 [47]. MBD6 preferentially associates with repressed rRNA genes, and this association is dependent on the de novo cytosine methyltransferase DRM2. In turn, DRM2-mediated DNA methylation is directed by siRNAs, the biogenesis of which relies on the RNA-dependent RNA polymerase RDR2, the Dicer nuclease DCL3, and the plant-specific DNA-dependent RNA polymerase (DdRp) Pol IV that synthesizes siRNA precursor transcripts. Interestingly, RDR2, DCL3, and the Argonaute protein AGO4 colocalize in the nucleolus along with rDNA-derived siRNAs, forming discrete foci that can be considered as siRNA processing centers [46]. Additionally, there is a feedback mechanism between DNA methylation and RNAi. The synthesis of siRNAs corresponding to the promoter region depends on DRM2, whereas the synthesis of siRNAs in the rDNA body requires the DNA methyltransferase MET1 that methylates cytosines in CG dinucleotides [74].

**Figure 1:**
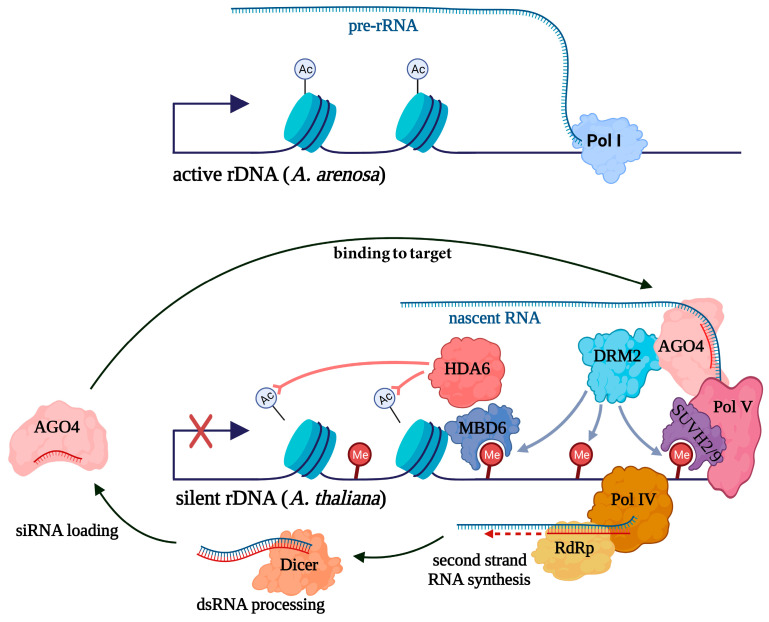
A simplified model for the mechanism of nucleolar dominance in a hybrid plant *A. suecica*. rDNA clusters from *A. arenosa* are actively transcribed and carry acetyl histone marks, while rRNA genes from *A. thaliana* are silent and have repressive characteristics such as DNA methylation (Me in red circles) and a lack of acetyl marks (Ac). This silencing is directed by siRNAs produced by the coordinated action of heterochromatin-specific RNA polymerase IV (Pol IV) and RNA-dependent RNA polymerase (RdRp) that generate dsRNAs used as substrates for Dicer. The resulting siRNAs are loaded into the AGO4-containing complex and guide it in a sequence-specific manner to nascent transcripts generated by another heterochromatin-specific RNA polymerase, Pol V. Binding of Pol V depends on SUVH2 and SUVH9 proteins interacting with methylated DNA. The AGO4 complex recruits DNA methyltransferase DRM2. Resulting DNA methylation through the methylcytosine-binding protein MBD6 presumably recruits histone modification enzymes including the histone deacetylase HDA6.

Overall, repression of the silent rDNA locus is maintained through a self-reinforcing loop similar to retrotransposons and other heterochromatic repeats in plant genomes (Figure 1). Pol IV is specifically recruited to heterochromatin and gives rise to transcripts that are used as templates for second-strand RNA synthesis by RDR2. The resulting double-stranded RNAs (dsRNAs) are processed by Dicer into siRNAs, which are loaded into an AGO4-containing effector complex. Generally, transcription silencing mechanisms via RNAi in eukaryotes are based on the interaction of the small RNAs with nascent transcripts in proximity to the genomic DNA locus producing them [39]. Thus, there is a paradox that transcription of a target is required for transcriptional silencing to occur. To do this, another plant-specific DdRp Pol V generates long non-coding RNAs that allow the recruitment of AGO4-containing complexes to target loci [75]. AGO4 recruits DRM2 to perform DNA methylation that in turn leads to histone deacetylation and the deposition of H3K9me2 modifications, repressing pre-rRNA synthesis by Pol I [76].

Thus, Pol IV specifically transcribes the silent rDNA locus, resulting in the formation of siRNAs that sustain the Pol V-dependent repression of these regions. In addition to providing RNA substrates for complementary siRNA binding, Pol V is thought to directly interact with AGO4, stabilizing it on the targeted loci [77]. Of note, Pol V association with chromatin requires DNA methylation [78]. It can be assumed that siRNA-AGO4 complexes cannot effectively interact with dominant rDNA locus because it lacks Pol V (Figure 1). Consequently, the silent rDNA cluster will be maintained in this repressed state, while the dominant one remains active. The initial determinants of which specific rDNA array undergoes repression are obscure but may be attributable to the local chromatin context of genomic regions adjacent to rDNA loci. Furthermore, given tissue and developmental variation in nucleolar dominance [69,70], the existence of the switching mechanisms potentially based on certain transcription factor binding can be hypothesized.

The implications of heterochromatin and small RNA pathways were also studied in relation to a phenomenon similar to nucleolar dominance observed in non-hybrid males of *D. melanogaster*. The male flies harbor two rDNA arrays, located on the X and Y chromosomes. The entire X chromosome rDNA cluster is typically silenced, while the Y chromosome’s rDNA array is expressed [66,79]. Although Su(var)3-9 H3K9 histone methyltransferase is pivotal in the silencing of the X chromosome rDNA, neither the siRNA nor piRNA pathways significantly affect this process [80]. Thus, although the role of heterochromatin in nucleolar dominance appears to be evolutionarily conserved, the participation of cis-acting siRNAs might rely on the reinforcing loop mechanisms of chromatin repression, characteristic of plants and some other organisms, but absent in flies. Nevertheless, examination of the potential role of small RNAs in the allelic inactivation of rDNA arrays in other animals, including human cells, presents an interesting avenue. Human cells have five nucleolar organizer regions (NORs), only some of which are normally active, while others remain completely silent [33,81]. This choice is likely established early in development through de novo methylation at rDNA promoter regions and then is maintained in somatic cells, potentially serving as a mechanism to regulate the number of available rDNA copies.

## 3. siRNAs Induce Selective Repression of 5S rRNA Gene Variants in Plants

The *A. thaliana* genome contains approximately 1000 tandemly organized copies of 5S rRNA genes in the pericentromeric heterochromatin regions of three chromosomes [82]. Transcription of a typical gene, approximately 0.5 kb in length, by canonical RNA polymerase III (Pol III) yields a 120 nt transcript. 5S rRNA gene activity can change during development, and their activation is accompanied by looping out of chromocenters [82]. There are two types of 5S rRNA genes, distinguished by several nucleotide substitutions: major genes, comprising 15–20% of 5S rDNA units and expressed in wild-type leaves, and minor genes, constituting up to 80–85% of 5S genes and typically repressed [83]. The transcriptional status of 5S rRNA genes depends on DNA methylation and RNAi mechanism. Treatment with DNA methylation inhibitor, 5-azacytidine, as well as mutations in genes encoding nucleosome remodeler DDM1, DNA methyltransferases, histone deacetylase HDA6, and AGO4 result in reduced DNA methylation and activation of minor 5S rRNA genes [82,84]. The presence of DCL3- and RDR2-dependent siRNA derived from 5S rDNA has also been shown [48]. Subsequent studies found at least two independent mechanisms responsible for maintaining the DNA methylation status of repressed 5S rRNA genes: the methylation of CG sites is maintained by the methyltransferase MET1 together with the chromatin remodeling factor DDM1, while asymmetrical CHH sites (where H is any base except G) undergo only de novo RNA-directed DNA methylation (RdDM) guided by siRNAs [49]. The mechanisms of MET1/DDM1-mediated methylation and RdDM are distinct but display a functional cross-talk. DNA methylation driven by MET1/DDM1 reduces the generation of siRNAs by limiting the formation of precursor transcripts. When DDM1 is deficient, there is an increase in siRNA production, activating RdDM, which, to some extent, compensates for the reduction in silencing.

In addition to siRNAs complementary to the 5S rRNA genes themselves, a fraction of siRNAs correspond to intergenic spacers (IGS). These siRNAs are required for the maintenance of silent chromatin in IGS regions. This prevents the production of aberrant elongated 5S rRNA transcripts, which can be synthesized when Pol III transcription, starting at the 5S rRNA gene promoter, passes through the 5S rRNA gene, and continues into the IGS [49]. Thus, siRNAs contribute to the quality control of 5S rRNA. Production of IGS siRNAs requires RdDM components, including Pol IV, RDR2, and Dicers. Interestingly, there are several subpopulations of IGS siRNAs, the biogenesis of which depends on different Dicer-like nucleases: DCL2, DCL3, and DCL4. This redundancy may enhance the sustainability of RNA-dependent silencing.

## 4. RNAi Pathways and rDNA Integrity

Two distinctive features of the rDNA locus—its repetitive structure and extensive transcription—make it susceptible to various types of DNA damage and spontaneous recombination events, resulting in the loss of portions of rDNA repeats. Hence, cellular mechanisms exist to safeguard the integrity of rDNA (reviewed in [21]). In various organisms, including mammals and flies, the local formation of heterochromatin structure on some rDNA units is thought to preserve the entire rDNA cluster stability. For instance, the depletion of the TIP5 protein, which mediates heterochromatin formation in mammalian cells, resulted in a significant loss of rDNA repeats [85]. The heterochromatic structure can physically prohibit the access of DNA damage factors [86]. It is also possible that components of heterochromatin, such as histone H3K9 methylation or HP1, can locally inhibit the activity of the recombination machinery. Interestingly, transcriptionally silent rRNA genes are predominantly lost upon Tip5 depletion [85], suggesting that an active rDNA transcription itself hinders the engagement of recombination complexes. *Drosophila* lacking Su(var)3-9, a histone methyltransferase crucial for heterochromatin formation, exhibited a reduced level of H3K9me2 repressive mark on rDNA and a rearrangement of the rDNA locus, as indicated by the formation of ribosomal extrachromosomal circularized DNA (eccDNA) [87], a hallmark of intrachromatid recombination accompanied by the loss of rDNA copy number [88]. A similar outcome was observed when Dicer-2, responsible for siRNA production, was lost [87]. This suggests that the RNAi pathway might play a role in promoting protective heterochromatin formation on rDNA repeats. However, knowledge about chromatin-based silencing induced by siRNAs in flies remains limited.

**Figure 2:**
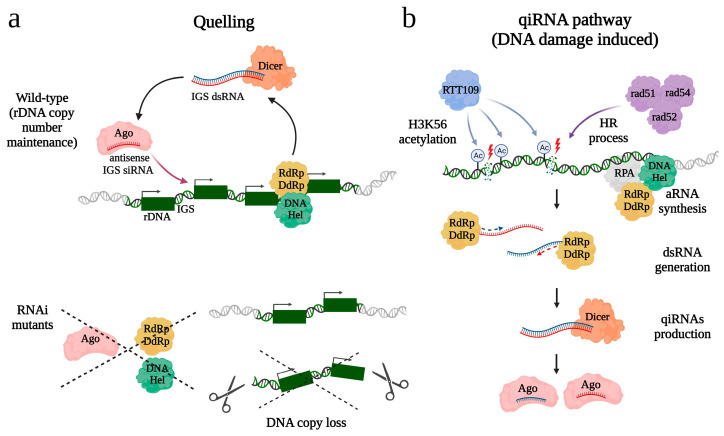
Proposed roles of RNAi pathways in rDNA integrity in *N. crassa*. The quelling (**a**) and qiRNA pathways (**b**) share three key components: QDE1 (RdRp/DdRp), which combines the activity of both RdRp and DdRp, an Argonaute protein QDE2 (Ago), and RecQ DNA helicase QDE3 (DNA Hel). (**a**) 20–25-nt siRNAs derived from transcripts of the IGS region control stability of rDNA copy number. (**b**) qiRNA production is induced upon DNA damage. Aberrant transcripts (aRNAs), synthetized by QDE1 (RdRp/DdRp) with participation of DNA Hel and a single-stranded DNA-binding protein RPA, are used for double-stranded RNA (dsRNA) production and subsequent processing into qiRNAs. Formation of qiRNAs requires H3K56 acetylation by histone acetylase RTT109 and involvement of the homologous recombination (HR) process with its main components rad51, rad52, and rad54.

In the filamentous fungus *Neurospora crassa*, the RNAi pathway known as quelling has also been implicated in maintaining the rDNA copy number [51]. siRNAs derived from IGS regions separating 45S rDNA repeats (i.e., 18S, 5.8S, and 28S) were found to be loaded into AGO protein QDE-2 (Figure 2a). A noticeable decrease in the rDNA copy number was observed upon the loss of key quelling components: QDE-2, QDE-1 (RdRp homolog), and QDE-3, a member of the RecQ DNA helicase family necessary for siRNA production. However, no significant change in the H3K9 methylation level of rDNA repeats was detected in these mutants. The authors suggested that the primary function of RNAi might be to silence bidirectional transcription from the IGS, which in budding yeast was shown to control recombination events in the rDNA locus [89]. Thus, RNAi pathways may play a role in preventing rDNA copy loss, either in conjunction with heterochromatin or through other mechanisms.

A similar RNAi pathway, also involving QDE 1-3 proteins, is activated in response to DNA damage in *N. crassa*, leading to the production of small RNAs known as qiRNAs [50] (Figure 2b). qiRNAs are characterized by a length of 20–21 nt, slightly shorter than siRNAs in *N. crassa*, and exhibit a strong preference for uracil at the 5’-end, a feature shared with piRNAs found in animals [50]. Over 85% of qiRNAs are derived from rDNA locus, including coding regions, IGS, and ITS [50]. A number of observations link qiRNA production to double-stranded break (DSB) repair at the rDNA locus. First, homologous recombination (HR) involved in DSBs repair is required for qiRNA production, as mutations leading to loss of HR cause a sharp decrease in the levels of detectable qiRNAs [52]. Second, the histone acetylase RTT109 and acetylation of histone 3 of the lysine residue at position 56 (H3K56Ac) contribute to both the DSBs repair process and qiRNA biogenesis [90]. Third, the synthesis of aberrant RNAs (aRNAs), acting as qiRNA precursors, and qiRNA formation depend on the RPA (Replication Protein A), a single-stranded DNA-binding protein, known to participate in HR [91,92]. Presumably, RPA recruits QDE-1, serving as both DdRp and RdRp, to its template and promotes transcription of aRNAs. It was hypothesized that qiRNA-mediated RNAi may be important to facilitate DNA damage repair including DSBs in the rDNA locus via inhibiting rRNA and protein synthesis [50]. It also cannot be excluded that qiRNAs can facilitate rDNA repair by direct recruitment of HR factors to DSBs, similar to what has been suggested for siRNAs derived from other loci in flies and mammals [93,94,95]. A pathway surprisingly similar to the *N. crassa* qiRNA pathway was reported in rice [96]. Treatment by DNA-damaging agents induced the production of rDNA-derived qiRNAs, and their precursors, aRNAs, in a manner dependent on RecQ DNA helicase and RdRp. Interestingly, DNA damage caused increased cell death and a more severe inhibition of root growth in both mutant lines than in the wild-type, suggesting that qiRNA biogenesis is crucial for DNA repair in rice.

As one of the most actively transcribed regions in the genome, rDNA should be protected from transcription-replication collisions that can lead to RNA:DNA hybrid formation and DNA damage with subsequent genomic instability. In dividing *Schizosaccharomyces pombe* cells, replication can collide with antisense rDNA transcription by RNA Polymerase II (Pol II) [97]. Dicer (Dcr1) was found to facilitate the release of transcribing Pol II from sites of replication stress within rDNA and, therefore, to resolve transcription-replication collisions [97]. Depletion of Dcr1 caused RNA:DNA hybrid formation in the rDNA and loss of a portion of rDNA repeats over several generations. Of note, siRNAs, produced by Dcr1, do not appear to play a role in this process.

In quiescent *S. pombe* cells, which do not undergo replication, depletion of Dcr1 leads to failure of Pol I release from rDNA [98]. In contrast to cycling cells, rDNA loss was not observed since quiescent cells utilize non-homologous end joining (NHEJ) instead of HR for DNA repair [99]. Nevertheless, Dcr1 depletion is accompanied by the accumulation of the DNA damage marker phosphorylated histone H2A and rDNA heterochromatinization, which is proposed to reduce cell viability. All key components of the *S. pombe* RNAi pathway—Ago1, Rdp1, and Dcr1—were essential for the survival of quiescent cells [98]. However, the involvement of small RNAs in Pol I release remains unclear.

## 5. Aberrant rRNAs Serve as Sources of Antisense Small RNAs That Can Induce rDNA Silencing

Across various organisms, it has been demonstrated that small RNAs, matching either antisense or both strands of rRNA sequences, can arise from erroneous or improperly processed rRNA molecules [53,56,57]. Normally, the quality control of rRNAs is upheld by multiple RNA surveillance mechanisms, which regulate the length of mature rRNAs, and degrade erroneous rRNA molecules [4]. When this machinery is compromised, anomalous rRNA transcripts accumulate and serve as substrates for the generation of antisense small RNAs, often referred to as risiRNAs. Antisense ribosomal siRNAs may also be synthesized in response to stressors, such as viral infections in plants [100], exposure to UV radiation, or cold shock in *C. elegans* [56]. These mechanisms rely on RdRp proteins, which utilize erroneous rRNA transcripts as templates to synthesize complementary small RNAs or double-stranded RNAs, processed into siRNAs by Dicer enzymes. Consequently, these mechanisms have been found in model organisms equipped with RdRp, including fission yeast [53], *N. crassa* [50], plants [101,102], and nematodes [56], but not in organisms lacking RdRp, such as the fruit fly *Drosophila* and mammals.

RNA exosomes play a pivotal role in processing and maturing rRNAs by trimming pre-rRNA intermediates and eliminating rRNA by-products [102,103,104]. In *Arabidopsis*, the loss of components of nuclear RNA exosome and cytoplasmic RNA decay processes triggers the production of aberrant rRNA-derived small RNAs [101,102]. Recently, the presence of RdRp/Dicer-dependent risiRNAs was observed in *Arabidopsis* following the loss of function of the HOT3 late-stage ribosome biogenesis factor [105]. risiRNAs produced in HOT3 mutants are complementary solely to the 3′ portion of 18S rRNA, aligning with HOT3’s role in the maturation of the 3′-end of 18S rRNA. The authors show that these risiRNAs do not affect the rRNA maturation process itself, being likely only by-products of rRNA metabolism.

In fission yeast *S. pombe*, the absence of a subunit in the TRAMP complex renders rRNAs susceptible to RdRp and Dicer activity, leading to the generation of antisense ribosomal siRNAs [53]. The evolutionarily conserved TRAMP complex is known to monitor the quality of various RNAs and polyadenylate junk rRNA transcripts, thus facilitating their degradation by RNA exosomes [106]. Subsequent studies showed that nitrogen starvation leads to the degradation of exosome/TRAMP, resulting in the enhancement of siRNAs and triggering heterochromatinization in rDNA regions [107].

**Figure 3:**
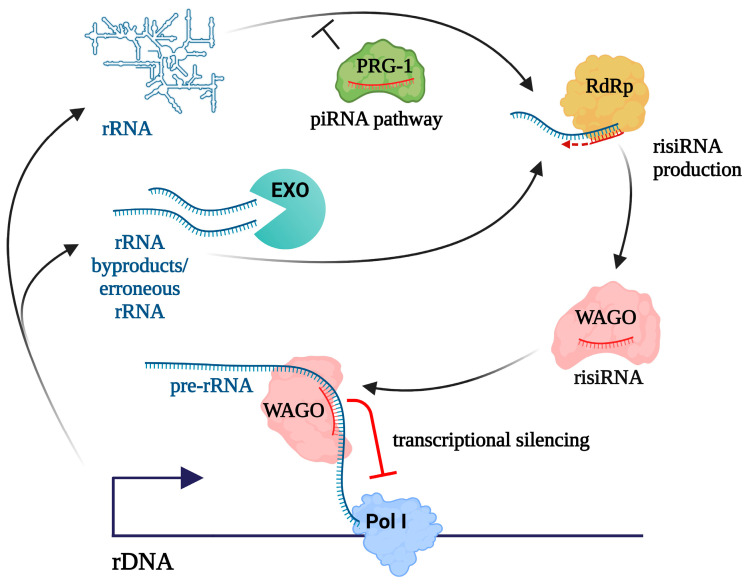
A model for antisense ribosomal small RNAs (risiRNA) production in *C. elegans*. risiRNAs are synthesized by RdRp from erroneous rRNAs and by-products of rRNA processing, which are normally eliminated by RNA exosomes and other RNA surveillance mechanisms (designated as EXO). piRNA pathway prevents risiRNA formation. risiRNAs are loaded into worm-specific Argonaute proteins (WAGO), including NRDE-3/WAGO-12, which can induce rDNA transcriptional silencing.

A thorough examination of the biogenesis of risiRNAs has been conducted in *C. elegans* [56]. In terms of their biogenesis, worm risiRNAs belong to the 22G-RNA class, one of the several small RNA categories in nematodes, named due to their propensity for a 5′ guanosine and their 22-nucleotide length. 22G-RNAs are produced by RdRp and are associated with worm-specific Argonaute proteins (WAGOs) [108]. risiRNAs, which are complementary to the 18S and 26S rRNA sequences, are present at basal levels in wild-type nematodes, but accumulate in RNA exosome mutants [109] or upon mutations affecting pre-rRNA processing and methyltransferases responsible for rRNA modifications [57]. Also, risiRNAs accumulate in the absence of DISL-2, a homolog of the cytoplasmic exoribonuclease, which degrades oligouridylated rRNA fragments [56]. Of note, rRNA fragments can be uridylated on their 3′ ends by RNA terminal uridyltransferases, making them preferred targets for decay. Thus, RdRp is thought to utilize improperly processed rRNA transcripts or 3′-tail-polyuridylated rRNA as templates for generating risiRNAs. These risiRNAs are loaded into WAGOs, including NRDE-3/WAGO-12, which are then translocated into the nucleus and reduce rDNA transcription level [56,109]. Therefore, misprocessed RNAs can act as a trigger for RNAi-mediated silencing of rDNA (Figure 3).

The biological significance of this feedback loop regulation remains enigmatic. Apparently, worm risiRNAs do not possess the ability to discriminate between damaged and functional rRNA molecules within the cell [56]. Thus, it is plausible that this mechanism is aimed at the indiscriminate reduction of rRNA synthesis in response to the accumulation of erroneous rRNAs. This may act as a phenoptotic phenomenon that can help eliminate mutants from the population [109]. However, a beneficial role for risiRNAs as responders to environmental stress cannot be excluded, given their increased presence during cold shock [56]. The resource-intensive process of ribosome biogenesis has been shown to be reversibly reduced or halted due to the stressful conditions in various organisms. Stress-induced rDNA repression can be initiated through diverse mechanisms, including the involvement of long non-coding RNAs PAPAS and IGS RNAs in mammals (for review, see [8]). Interestingly, in *S. pombe*, heterochromatinization and transcriptional silencing of rDNA serve as a vital adaptation to glucose starvation, and this process requires the RNAi pathway [110].

## 6. Implications of the piRNA Pathway in rRNA Regulation

The most extensively investigated role of the piRNA pathway is the repression of transposons and other selfish genetic elements residing within animal genomes. From a broader perspective, the piRNA pathway serves as a mechanism, distinguishing between self and nonself nucleic acids that are reflected by its functions in viral defense, and licensing of self transcripts in the *C. elegans* germline (for review, see [44]). Notably, the piRNA pathway was found to suppress risiRNA production in nematodes [111,112]. This finding provided an explanation for the long-known phenomenon of transgenerational sterility: worms lacking the piRNA pathway components are superficially normal but progressively become infertile after approximately 17 generations. The observed sterility in piRNA-deficient animals was correlated with the hyper-accumulation of 22G risiRNAs. Mutations in the siRNA pathway and uridylation machinery, as well as the addition of extra rDNA units, rescued this sterility phenotype [111]. These results suggest the piRNA pathway prevents the accumulation of risiRNAs to the deleterious levels that cause sterility through rDNA silencing (Figure 3). Since rRNA has been shown to interact with piRNA complexes [113], it is plausible that they physically block the association of rRNA with the 22G-RNA machinery [112]. Hence, Piwi-piRNA complexes may play a protective role for rRNA. Interestingly, the accumulation of rRNA fragments was observed in *Drosophila* ovaries with mutations in the piRNA pathway [114], which can be hypothetically attributed to the increased accessibility of rRNA to certain nucleases in the absence of the piRNA machinery.

Studies in *Drosophila* point to another potential function of the piRNA pathway linked to rRNA. Up to 70% of fly rRNA genes contain insertions of specific retrotransposons R1 and R2 within the 28S rRNA sequence [115]. These elements can be transcribed solely under the control of the rDNA promoter and subsequently excised from pre-rRNA. Typically, interrupted rDNA units are transcribed orders of magnitude less than intact rRNA genes [35,116]. The repression of interrupted rDNA units usually relies on poorly understood molecular mechanisms unrelated to small RNA silencing [35,117]. Intriguingly, interrupted rDNA units are upregulated in response to heat shock in *Drosophila* ovarian cells [118,119]. Furthermore, heat shock also leads to the accumulation of nuclear piRNA-binding protein Piwi in the nucleolus [119]. Expression levels of interrupted rDNAs were found to be significantly higher during heat shock when Piwi is knocked down compared to heat shock with intact Piwi. Thus, Piwi appears to restrain the expression of interrupted rDNAs during cellular stress, which potentially weakens their control by other repression pathways. *Drosophila* Piwi is unable to conduct endonucleolytic slicer activity and is known to induce chromatin-based transcriptional repression on its targets [120,121,122,123]. Therefore, it is plausible that Piwi may influence rDNA transcription rather than the stability of RNAs produced by interrupted rDNA units. The ways by which Piwi-piRNA complexes achieve this regulation remain to be determined.

## 7. Potential Mechanisms of rDNA Silencing by RNAi in Animals

Data from various eukaryotes show that small RNAs are capable of regulating rDNA at the transcriptional level. Generally, transcription silencing mechanisms via RNAi are based on the interaction of the AGO-small RNA complex, such as the RITS complex in *S. pombe*, with nascent transcripts [124]. This interaction leads to the recruitment of transcription repressors or chromatin factors, which then deposit repressive histone marks and induce DNA methylation in certain organisms. How can these mechanisms be adapted for rDNA regulation in animals? In *C. elegans*, risiRNAs have been shown to repress transcription elongation of Pol I by decreasing its occupancy downstream of the RNAi-targeted site [109], similar to observations for Pol II transcripts during nuclear RNAi targeting protein-coding genes [125]. However, in contrast to Pol II-dependent targets, significant changes in H3K9 and H3K27 methylation at the rDNA locus in the presence of risiRNAs were not observed [109]. Interestingly, canonical heterochromatin factors only minimally affect the repression of individual rDNA units in *Drosophila* [35,117]. Instead, their silencing is heavily influenced by the SUMOylation (covalent attachment of Small Ubiquitin-like Modifier) of some unidentified nucleolar targets [117]. While these findings are fragmentary, it is tempting to speculate that the mechanisms of epigenetic silencing, including those potentially involving RNAi-based regulation, may exhibit distinctive characteristics in the context of rDNA loci.

In mammals, Piwi-piRNA complexes, assisted by accessory proteins, induce both DNA methylation and repressive histone modifications on target loci [126,127,128,129]. Multiple pieces of evidence also indicate that small RNAs associated with AGO1 and AGO2 proteins in human cells are able to induce transcriptional silencing or sometimes activation of target genomic sequences, but the underlying mechanisms remain under-investigated (reviewed in [130]). A recent report demonstrates that in quiescent mammalian cells, AGO2 accumulates in the nucleus, where it co-transcriptionally represses transposable elements [131]. Although there is abundant information about small RNAs produced from the mammalian rDNA loci, most of them are assumed to regulate targets outside the nucleolus (reviewed in [45]), and little is known about their potential influence on rDNA itself. Dicer has been found within the nucleolus, interacting with rRNA genes in human cells [132], which might, however, reflect the fact that it facilitates the processing of pre-rRNA precursors [133]. Another report demonstrated the interaction of human AGO2 with rDNA chromatin [134]. These interactions were drastically reduced when cells were briefly treated with actinomycin D, impairing Pol I transcription. Knockdown of AGO2 resulted in a statistically significant increase in the overall rRNA synthesis rate but did not affect the recruitment of the Pol I transcription factor UBF. These results hint at the potential for the regulation of rDNA transcription by small RNAs in humans.

## 8. Conclusions and Perspective

In this review, we have compiled data from diverse eukaryotic organisms, shedding light on the roles of small RNAs in various molecular processes related to rRNA regulation. These functions encompass chromatin-based silencing of rDNA arrays, the maintenance of rDNA cluster integrity, and rDNA repression in response to stressful conditions.

Mammalian cells produce a plethora of small RNAs from rDNA loci. Although most of these small RNAs are derived from mature rRNA molecules, a fraction of antisense ribosomal RNAs capable of regulating rRNA expression has also been observed [60]. Recent studies have convincingly shown the fundamental ability of mammalian siRNAs to guide nuclear processes such as the regulation of transcription and splicing [130]. From this perspective, it is intriguing to explore the potential role of mammalian antisense ribosomal small RNAs in nuclear mechanisms related to rDNA. Potential avenues of investigation may include evaluating mechanisms that maintain rDNA copy numbers, which remain largely unknown in mammals. This becomes particularly important in the context of aging and cancer cells, which are characterized by pronounced rDNA instability [135,136]. It is conceivable that these mechanisms might rely on the ability of nuclear RNAi pathways to establish recombination-resistant chromatin in rDNA or on the functions of small RNAs in DNA damage repair. Additionally, the involvement of small RNAs in rRNA quality control cannot be excluded, given the regulatory potential of small RNAs to select target transcripts in a sequence-specific manner.

## Data Availability

Not applicable.
